# DNA methylation patterns in newborns exposed to tobacco in utero

**DOI:** 10.1186/s12967-015-0384-5

**Published:** 2015-01-27

**Authors:** Carmen Ivorra, Mario F Fraga, Gustavo F Bayón, Agustín F Fernández, Consuelo Garcia-Vicent, F Javier Chaves, Josep Redon, Empar Lurbe

**Affiliations:** Pediatrics Department, Consorcio Hospital General, University of Valencia, Valencia, Spain; CIBER Fisiopatología Obesidad y Nutrición (CB06/03), Instituto de Salud Carlos III, Madrid, Spain; IUOPA Cancer Epigenetics Laboratory, Department of Immunology and Oncology, Centro Nacional de Biotecnología/CNB-CSIC, Instituto Universitario de Oncología del Principado de Asturias (IUOPA), HUCA, Universidad de Oviedo, Oviedo, Spain; Hospital Clínico de Valencia-INCLIVA Valencia, University of Valencia, Valencia, Spain; CIBER de Diabetes y Enfermedades Metabólicas (CIBERDEM), Instituto de Salud Carlos III, Madrid, Spain

**Keywords:** Tobacco, Newborns, DNA methylation, Adrenomedullin gene

## Abstract

**Background:**

Maternal smoking during pregnancy is a major risk factor for adverse health outcomes. The main objective of the study was to assess the impact of in utero tobacco exposure on DNA methylation in children born at term with appropriate weight at birth.

**Methods:**

Twenty mother-newborn dyads, after uncomplicated pregnancies, in the absence of perinatal illness were included. All mothers were healthy with no cardiovascular risk factors, except for the associated risks among those mothers who smoked. Umbilical cord blood and maternal peripheral venous blood were collected and an epigenome-wide association study was performed using a 450 K epigenome-wide scan (Illumina Infinium HumanMethylation 450BeadChip) with adjustment to normalize the DNA methylation for data cell variability in whole blood.

**Results:**

The maternal plasmatic cotinine levels ranged from 10.70-115.40 ng/ml in the exposed group to 0-0.59 ng/ml in the non-exposed group. After adjusting for multiple comparisons in 427102 probes, statistically significant differences for 31 CpG sites, associated to 25 genes were observed. There was a greater than expected proportion of statistically-significant loci located in CpG islands (Fisher’s exact test, p = 0.029) and of those CpG islands, 90.3% exhibit higher methylation levels in the exposed group. The most striking and significant CpG site, cg05727225, is located in the chromosome 11p15.4, within the adrenomedullin gene.

**Conclusions:**

In utero tobacco exposure, even in the absence of fetal growth restriction, may alter the epigenome, contributing to global DNA hypomethylation. Therefore, DNA status can be used as a biomarker of prenatal insults. Considering the possibility to reverse epigenetic modifications, a window of opportunity exists to change the programmed chronic disease.

## Background

Maternal smoking during pregnancy is a major risk factor for adverse health outcomes in children. Recent evidence suggests that it has consequences that are not only immediate, such as low birth weight, but it also leads to long-term risk for obesity, type 2 diabetes, elevated blood pressure and neurobehavioral defects in children [1-4]. Despite public health warnings, in Spain 16% of pregnant women are smokers [4], implying that a large number of offspring are at risk. Even though it is clear that there are adverse health consequences, the mechanisms behind the relationship between in utero tobacco exposure and its effects are not well understood.

Changes to DNA methylation can occur throughout life, but much of the epigenome is established during embryogenesis and early development of the fetus [5]. Epigenome changes that are mitotically stable and heritable are also sensitive to environmental stimuli. Then, the epigenetic process provides a window through which, the genes that are involved in the lifelong effects of in utero insults, can be identified [6]. DNA methylation in different regions can have different effects. While in gene promoter regions, overall those located in cytosine–guanine dinucleotide (CpG) islands, reduce the gene transcription, methylation in the body of genes can increase the transcription [7].

Recent studies in human populations have demonstrated that tobacco exposure may induce epigenetic alterations, specifically by altering patterns of DNA methylation [8]. Although largely studied in adult smokers, few human studies have examined epigenetic alterations in relation to maternal smoking during pregnancy and reported it to be associated with global methylation of leukocyte DNA [9-11]. These studies have used global methylation with a [3H]-methyl acceptance assay [9,10] or differential methylation at CpG sites [11]. Maternal smoking during pregnancy has also been associated with global and CpG-site specific methylation in human placenta [11] as well as in buccal cells from children [12].

The objective of the present study was to assess the impact of in utero tobacco exposure on DNA methylation in children born at term and appropriate for gestational age. The epigenome-wide association study was performed on umbilical cord blood, using a global methylation assay and a 450 K epigenome-wide scan with the Houseman adjustment method [13] to normalize the DNA methylation for data cell variability in whole blood. Cotinine levels in umbilical cord blood were the markers of in utero tobacco exposure.

## Methods

### Study participants

Subjects were recruited from those born in the Hospital General Universitario of Valencia, Spain between January and December 2011. Mothers were invited to participate at the time of their admission to the Hospital for delivery. Exclusion criteria were missing information about the pregnancy. Twenty newborns and their mothers fulfilled the criteria of being of European origin, born at term (gestational age ≥37 weeks), and appropriate for gestational age, defined as weight at birth between 10th and 90th percentile [14]. All subjects were born from uncomplicated pregnancies in the absence of perinatal illness, and gave informed parental consent to participate. Additional inclusion criteria were the concordance between the questionnaire and the cotinine levels. Mothers completed a questionnaire that included the details of their smoking habits. Eligibility by exposed status was determined through maternal self-report and confirmed by blood cotinine levels. Subjects were included in the exposed group from those who were daily smokers before pregnancy and maintained the same habit throughout the three trimesters. All mothers were healthy with no cardiovascular risk factors, except the associated risks among those mothers who smoked. Additionally, subjects were selected for the non-exposed group based on sex distribution and mothers’ age.

In utero tobacco exposure was assessed by cotinine determination in plasma from maternal peripheral blood and in the infants’ umbilical cord blood. Umbilical cord blood samples were obtained from the clamped umbilical cord immediately after delivery. Samples from the mothers were drawn 24 h after delivery. The samples and collected data were stored according to the directives dictated by the law of Biomedical Investigation of 2007 (Law 14/2007). The study was approved by the review board of the Hospital General Universitario de Valencia and was carried out in accordance with the Declaration of Helsinki. Plasma and DNA from umbilical cords of the newborns and from peripheral veins from the mothers, as well as the collected data, were stored according to the directives dictated by the law of Biomedical Investigation of 2007 (Law 14/2007) and all applicable rules.

Tobacco exposure was measured using an immunoassay for cotinine (Salimetrics, SPK 1-2002-5) according to the manufacturer’s recommendations. The minimum concentration that can be distinguished is 0.05 ng/ml. Newborn groups were created according to cotinine levels in umbilical cord; higher than 10 ng/ml was qualified as exposed to in utero tobacco and lower than 1 ng/ml was considered as non-exposed to in utero tobacco.

### Sample collection, DNA extraction, quantification and quality check

Umbilical cord blood and maternal peripheral venous blood were collected in EDTA-tubes, centrifuged to yield plasma, stored at -80°C and thawed before use. Genomic DNA was extracted from venous umbilical cord blood with the RealPure kit (REALPURE, REAL, DURVIZ, Ref: RBMEG01) and was quantified with the Nanodrop-2000C Spectrophotometer. A DNA quality check was performed with Quant-iT PicoGreen dsDNA reagent.

### Global DNA methylation assay

The global DNA methylation levels in umbilical cord blood samples were obtained with an ELISA commercial kit (5-mC DNA ELISA Kit, Zymo Research, D5325). Two hundred nanograms (ng) of DNA were diluted with 250 μl of 5-mC Coating buffer lysis and incubated at 98°C for 5 minutes. After denaturation, DNA were transferred to the plate and incubated at 37°C for one hour. The samples were incubated with capture and detection antibodies and absorbance was read at 450 nanometers. Quantification of global DNA methylation was obtained from calculating the amount of methylated cytosines in the sample (5 mC) relative to global cytidine (5 mC + dC) in a standard curve following positive control that had been previously methylated. All samples were analyzed in duplicate.

### Illumina 450 K methylation data analysis

Genomic DNA (one μg) from each subject was treated with sodium bisulfite using the EZ DNA Methylation kit (ZYMO RESEARCH CORP, Irvine, CA, USA) according to the manufacturer’s recommendations. The incubation profile was 16 cycles at 95°C for 30 s, 50°C for 60 min and a final holding step at 4°C (incubation conditions recommended when using the Illumina Infinium Methylation Assay). The methylation assay was performed on 4 μl of converted bisulfite DNA using the Illumina Infinium HumanMethylation 450BeadChip following the Infinium HD Methylation Assay protocol.

For HumanMethylation 450 K array analysis, the sample was divided in 2 groups of 10 samples with the same proportion of samples corresponding to the exposed and non-exposed groups and samples were located in the chip by random distribution, in order to avoid the batch or localization effects. Methylation profiling of the samples was carried out using Illumina Infinium HumanMethylation450 BeadChip Kit (Illumina). Instead of using Illumina's GenomeStudio software, raw IDAT files were obtained and further preprocessed using the R/Bioconductor*minfi* package [minfi]. Red and green channel intensities were obtained from the raw IDAT files. Detection p-values for each probe were also computed and used in order to discard those probes with low quality. A probe was discarded if at least 2 of its detection p-values were over 0.01.

The normalization step used the SWAN method to correct the differences that appeared due to the two different probe designs present in the Infinium HumanMethylation450 [15]. A set containing both M (methylated) and U (unmethylated) signals was obtained as a result of these two normalization steps.

Probes contained in the set with the M and U signals were annotated in a separate step. Probes where at least two samples had detection p-values over 0.01 were filtered out. Each probe was initially labelled with the information included in the Illumina Manifest file which was directly related to the location-dependent properties of the probe, including the chromosomal location of the probe, percent GC and source genomic sequence for the probe, among other fields. All the annotation data needed for the posterior analyses was dynamically evaluated using the *rtracklayer* package from R/Bioconductor [rtracklayer] for accessing the UCSC Browser database [ucsc].

Nearest gene ID, symbol and the distance from the probe to the nearest TSS were obtained from the *TxDb.Hsapiens.UCSC.hg19.knownGene* [txdb] and *BSgenome.Hsapiens.UCSC.hg19* [bsgenome] packages from R/Bioconductor. CpG Island status was evaluated with respect to R. Irizarry's automatically-generated description of CpG Island regions, which is by default included in the *FDb.InfiniumMethylation.hg19* package from R/Bioconductor. Shores were defined as 2 kb regions flanking CpG Islands, and Shelves as 2 kb regions following the respective upstream and downstream Shores.

From the M and U signals, both beta-values were generated (which are easier to interpret from a biological point of view) and M-values (a transformation of beta values to achieve homoscedasticity [mvalues]).

All CpG loci on X and Y chromosomes were excluded from the analysis to avoid sex-specific methylation bias. Probes that were found to be cohybridated with probes in the sexual chromosomes [16] were also removed. The information from the SNP137Common track from the UCSC browser was used [17], in order to remove those probes with an SNP located inside their 2 bp central region.

### Data analysis

Differences in global DNA methylation were sought by using Student’s t-test for independent samples. A linear model approach was performed to identify individual CpG loci showing differential methylation values associated with in utero tobacco exposure. Significant differentially methylated probes were determined by the moderated t-test implemented in the R/Bioconductor package "limma" [18]. P-values were adjusted by controlling the FDR (using the Benjamini-Hochberg method). Probes with adjusted p-values under a 0.05 significance level were selected. Samples were clustered according to their Euclidean Distance, and a complete linkage clustering method was used for generating the dendrograms.

Methylation data was adjusted for white blood cell heterogeneity using the method described in [13]. Since unfractioned PBLs from whole blood was collected, the methylation profile in PBLs represents the aggregate methylation profile of a complex cellular mixture. Thus, even small changes in percentage methylation may indicate considerable differences in underlying cell populations. To address this limitation, a novel statistical methodology, created by Houseman and co-workers [13] was employed. This method was used to quantify the proportion of total variability in cord blood DNA methylation explained by estimated immune cell composition between subjects. In order to feed this method, the original Illumina 27 k database of purified white blood cell subtypes was used, included in the authors' original implementation of the algorithm.

### Adrenomedullin quantification

The adrenomedullin (ADM) concentration in plasma from umbilical cord samples was determined using a high sensitivity ELISA kit for human Adrenomedullin, (Uscn Life Sicence, Cat. No. CEA220Hu), according to the manufacturer’s protocol. All samples were analyzed in duplicate. The minimum detectable dose of ADM is 4.77 pg/mL.

### Data access

The HumanMethylation450 BeadChip data sets from this study have been submitted to the NCBI Gene Expression Omnibus (GEO; http://www.ncbi.nlm.nih.gov/geo/) under accession number GSE64316.

## Results

### General clinical and anthropometric characteristics of the study population

The general characteristics of the newborns and their mothers included in this study are in Table 1. Two groups of newborns were considered, exposed (n = 10, five females) and non-exposed (n = 10, five females) to in utero tobacco. The maternal plasmatic cotinine levels ranged from 10.70-115.40 ng/ml in the exposed group to 0-0.59 ng/ml in the non-exposed group (see Table 1). In exposed and non-exposed mothers no differences were observed in BMI at the beginning of pregnancy (20.8 ± 4.4 vs 23.3 ± 5.1, p = 0.26) and BPs (110/67 ± 7.3/6.9 vs 106/66 ± 6.8/5.9, p = 0.21 and 0.61, respectively) as well as BP at the time of labour (126/69 ± 4.6/4.2 vs 125/73 ± 5.8/5.2, p = 0.56 and 0.13, respectively). When the general characteristics from the two groups were analyzed, no significant differences were observed in maternal age, gestational age, length, blood pressure and heart rate between exposed and non-exposed newborns. However, birth weight was significantly lower among exposed and non-exposed newborns (3361 g ± 272 *vs* 3713g ± 147; p-value = 0.0021) although none were qualified as small for gestational age, birth weight <10^th^ percentile [14].

**Table 1:** **General characteristics of mother and newborns grouped according to in utero tobacco exposure**

**Variable**	**Exposed (n = 10)**	**Non-exposed (n = 10)**	**p-value**
**Maternal Characteristics**			
Maternal age (years)	31.3 ± 6.8	29.9 ± 7.4	0.681
Maternal Cotinine (ng/ml)	58.30 ± 34.23	0.05 ± 0.16	<0.001
**Newborn Characteristics**			
Gestational age at delivery (wk)	38.9 ± 1.45	38.9 ± 0.74	1.000
Sex (male/female)	5/5	5/5	
Birth weight (g)	3361 ± 272.2	3713 ± 147.3	0.0021
Length (cm)	49.9 ± 1.47	49.5 ± 1.58	0.928
Systolic blood pressure (mmHg)	74.43 ± 13.62	74.60 ± 13.72	0.979
Diastolic blood pressure (mmHg)	47.73 ± 13.42	44.57 ± 12.69	0.594
Heart rate (bpm)	119.40 ± 11.74	119.43 ± 10.32	0.963
Umbilical cord blood cotinine (ng/ml)	65.47 ± 36.71	0.115 ± 0.24	<0.001

The values are expressed as mean ± SD; p-value, statistical significance of the differences among groups.

### Global DNA methylation

**Figure 1:**
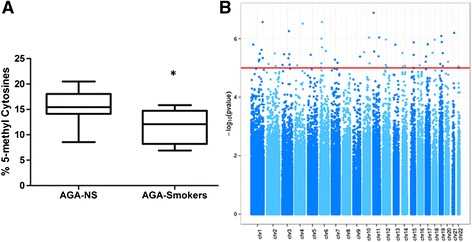
**Global DNA Methylation. A)** Global DNA methylation index in cord blood DNA of 10 newborns exposed to in utero tobacco, and 10 newborns non-exposed to in utero tobacco (Box plots). **B)** Manhattan plot for methylation status in umbilical cord blood DNA from newborns exposed or non-exposed to in utero tobacco. The vertical axis indicates (-log_10_ transformed) observed p-values and the horizontal threshold indicates the significance level (*p* = 1x10^−5^).

Global DNA methylation status in whole blood was compared between the groups using a 5-methyl Cytosine kit. A significantly lower global methylation was found in the exposed group (see Figure 1A).

### Epigenome-wide analysis by 450 K platform

Epigenome-wide DNA methylation profile was determined using Illumina Human BeadsChips 450 K. This array enables site-specific methylation status of 485577 CpG sites across the human genome. It contains probes to detect methylated and unmethylated sequences and data from probes are used to calculate a β-value between 0 and 1 (equivalent to 0%-100% methylation). After quality control (3457 probes were excluded), methylation data from 482120 CpG sites were available. The methylation analysis of 10916 probes located in X/Y chromosomes is perfectly correlated with gender. After this verification, all CpG loci on X and Y chromosomes were excluded from the analysis to avoid sex-specific methylation bias. Additionally, the CpG sites containing a common SNP or co-hybridizing probes in sexual chromosomes were removed to avoid sex-specific methylation bias and biases related to genetic variability respectively. The final dataset comprised methylation data for 427102 autosomal CpG loci for analysis in 20 samples.

**Figure 2:**
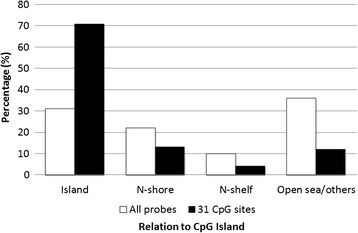
**Location of the 31 CpG sites differentially methylated compared to all CpGs on the methylation array (all probes).** Methylation sites were categorized in groups according to their location.

To identify individual CpG loci whose methylation status was associated with prenatal tobacco exposure, the differential locus-specific patterns were examined. After adjusting for multiple comparisons using a FDR 0.05 in 427102 probes, statistically significant differences for 31 CpG sites, associated to 25 genes were observed (5 genes present 2 sites with differential methylation and one of the sites is located in an intergenic region). Table 2 displays all CpG sites with a significant differential methylation between exposed and non-exposed groups. A visual representation of the genome-wide distribution of the significant differentially-methylated CpG sites of exposed vs. non-exposed is represented as Manhattan plot in Figure 1B. We found a greater proportion of loci located in CpG islands compared to those outside of these regions (Fisher’s exact test, p = 0.029) (Figure 2). Those methylation sites located in CpG islands showed higher methylation levels (90.3% of the sites) in the exposed group. Only 3 CpG sites were hypomethylated in the exposed group.

**Table 2:** **Significantly differentiated methylated CpG sites of newborns exposed to inutero tobacco compared to non-exposed**

**CpG**	**Chromosome**	**Gene**	**Logfold change**	**p-value**	**Adjusted p-value**	**Delta beta**	**Rank**	**CpG_location**
cg05727225	11p15.4	*ADM*	0.3474	1.32xe-07	0.03282	0.0432	1	CpG island
cg00387170	6p21.1	*FOXP4*	0.4894	2.71xe-07	0.03282	0.0740	2	CpG island
cg13907959	1q42.13	*HIST3H2A*	−0.4731	2.72xe-07	0.03282	−0.0409	3	CpG island
cg02738677	1q42.13	*HIST3H2A*	0.4894	5.56xe-07	0.0385	0.0685	4	Non-CGI
cg04402350	4q31.22	*ZNF827*	0.5450	3.07xe-07	0.0328	0.0676	5	CPG island
cg25783241	21q22.3	*AIRE*	0.2620	6.34xe-07	0.0385	0.0435	6	CPG island
cg04177517	19q13.11	*GPATCH1*	0.3278	8.06xe-07	0.0385	0.0778	7	CGI-Shore
cg12331332	10q25	*CASP7*	0.3305	9.07xe-07	0.0385	0.0390	8	CPG island
cg24962978	11q23.3	*RPS25*	0.5570	9.13xe-07	0.0385	0.0742	9	CPG island
cg15264752	6p21.3	*DDAH2*	0.4845	1.00xe-06	0.0385	0.0526	10	CPG island
cg23240961	12q13.3	*NDUFA4L2*	0.5615	1.06xe-06	0.0385	0.0666	11	CPG island
cg27561567	17q11-qter	*CLTC*	0.2552	1.08xe-06	0.0385	0.0623	12	Non-CGI
cg08533403	19q13.2	*PLD3*	0.4951	1.31xe-06	0.0403	0.0750	13	CPG island
cg03621881	15q24	*CELF6*	0.4745	1.32xe-06	0.0403	0.0522	14	CPG island
cg12556991	17q21	*RPL27*	0.5845	1.55xe-06	0.0403	0.0528	15	CPG island
cg13549152	1p34.3	*AKIRIN1*	0.2472	1.57xe-06	0.0403	0.0494	16	CGI-Shore
cg10454248	13q14.3	*FAM124A*	0.5438	1.60xe-06	0.0403	0.0532	17	CPG island
cg09048334	17q21	*RPL27*	0.3934	1.98xe-06	0.0472	0.0919	18	CPG island
cg00093900	1q32.1	*PPP1R15B*	0.3873	2.39xe-06	0.0494	0.0363	19	CPG island
cg02192300	13q14.3	*FAM124A*	0.3810	2.63xe-06	0.0494	0.0446	20	CPG island
cg03529555	11p15.1	*NAV2*	−0.4831	2.72xe-06	0.0494	0.0642	21	Non-CGI
cg26547359		*HS3ST5*	−0.4682	2.89xe-06	0.0494	0.0524	22	CPG island
cg01249134	11p15.1	*NAV2*	0.3364	3.05xe-06	0.0494	0.0758	23	CGI-Shelf
cg07455406		*RSU1*	0.2540	3.08xe-06	0.0494	0.0624	24	CPG island
cg12271800	19q13.43	*ZBTB45*	0.4554	3.10xe-06	0.0494	0.0473	25	CPG island
cg25932748	2q24.1	*KCNJ3*	0.5870	3.18xe-06	0.0494	0.0666	26	CGI-Shore
cg17423711	1q24.2	*POU2F1*	0.3053	3.24xe-06	0.0494	0.0260	27	CPG island
cg23332582	3q23	*SPSB4*	0.3668	3.36xe-06	0.0494	0.0673	28	CPG island
cg09352908	5q12	*PDE4D*	0.4439	3.38xe-06	0.0494	0.0637	29	CGI-Shore
cg22078451	5q12	*PDE4D*	0.5968	3.53xe-06	0.0494	0.0641	30	CPG island
cg20916068			0.4586	3.58xe-06	0.0494	0.0986	31	Non-CGI

The most striking and significant CpG site, cg05727225, (adjusted p-value = 0.03282, fold change = 0.3474) is located in the chromosome 11p15.4, within the ADM gene. Three more CpG sites are located in the 11 chromosome: cg24962978 associated to *RPS25* gene, and cg03529555 and cg01249134 loci associated to *NAV2* gene. On chromosome 1, 4 CpG sites were found in which methylation is increased in the exposed group associated to *HIST3H2A*, *AKIRIN1*, *PPP1R15B* and *POU2F1* genes, and one loci hypomethylated in *HIST3H2BB* gene in the exposed group. Other genes in which methylation is statistically significant between the study groups are *FOXP4, ZNF827, AIRE, GPATCH1, CASP7, MIR3656, TRAPPC4, DDAH2, NDUFA4L2, LINC00669, C19orf47, PLD3, CELF6, RPL27, AKIRIN1, FAM124A, HS3ST5, RSU1, ZBTB45, KCNJ3, SPSB4* and *PDE4D*. Locations, p-values, rank and fold changes are provided in Table 2.

**Figure 3:**
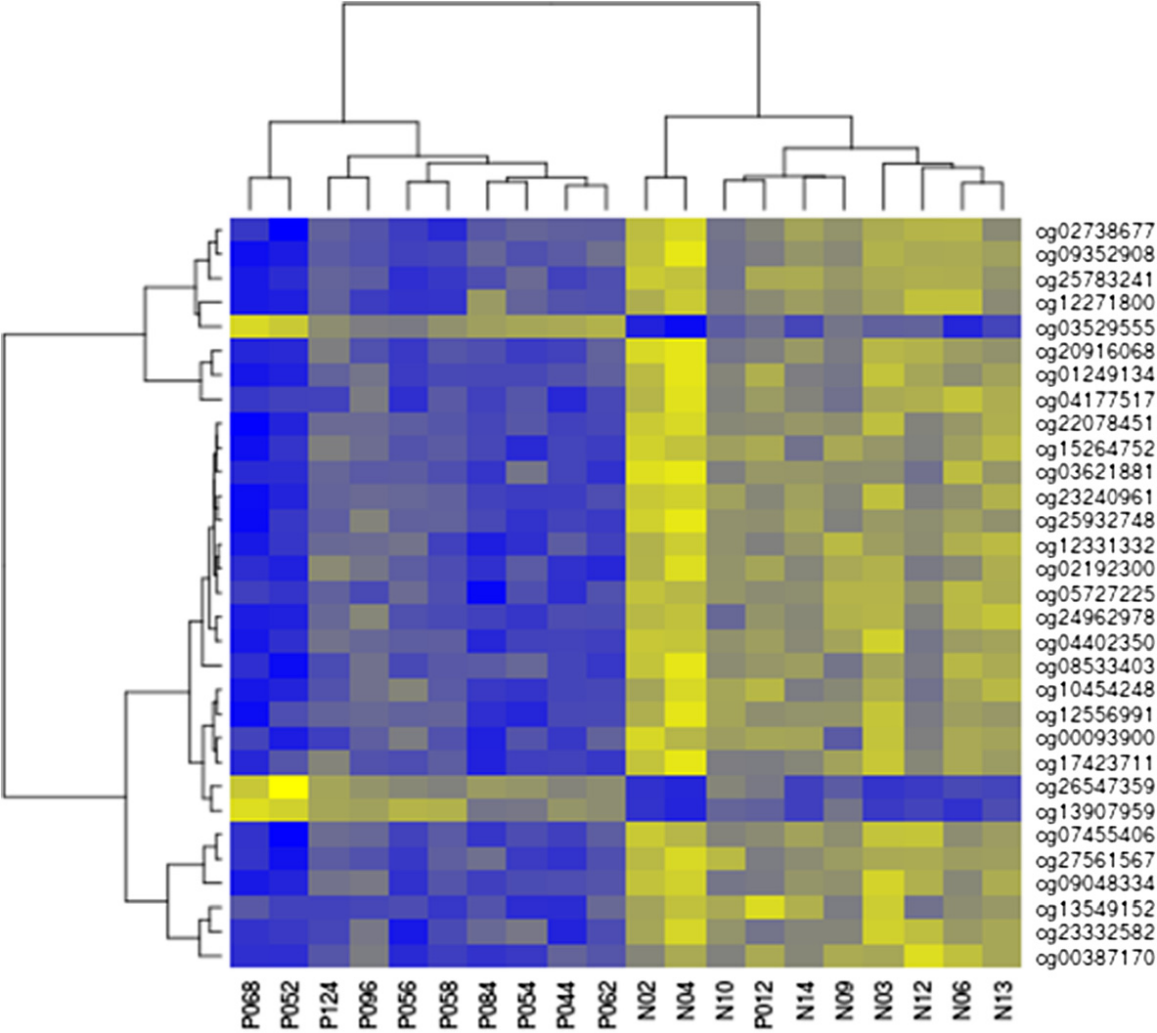
**Hierarchical clustering heat map including the CpG sites with significant differential methylation between exposed and non-exposed newborns.**

Unsupervised clustering of samples using the methylation signals of the 31 differentially methylated CpG sites revealed two main clusters: one cluster comprising the exposed newborns and the other containing the non-exposed newborns (Figure 3). Interestingly the samples displaying higher differential methylation correspond to the newborns that were more exposed to tobacco compounds (N2, N4 and N13).

### Adrenomedullin

Adrenomedullin in the cord blood of the exposed and non-exposed groups was assessed and the mean levels were 10.0 ± 2.2 and 10.2 ± 3.9 pg/mL respectively. Furthermore there were no significant differences in ADM values in the umbilical cord status between groups adjusted for birth weight. When the groups were divided into those with lowest (n = 5 in each group) and highest (n = 5 in each group) birth weights, in the 10 smaller newborns of the exposed and non-exposed groups the mean was 10.3 ± 2.7 and 7.6 ± 1.9 respectively (p = 0.72). In the 10 larger newborns of the 2 groups the mean was 9.7 ± 1.8 and 12.6 ± 3.5, respectively (p = 0.58).

## Discussion

The impact of in utero tobacco exposure on DNA methylation is of great interest. Patterns of DNA methylation, which have a role in switching genes on and off, are substantially established during embryogenesis and fetal development. In this way, the findings of the present study provide additional information on the issue. Inappropriate for gestational age newborns, born at term, DNA global methylation of umbilical cord blood was lower in those exposed to in utero tobacco as compared to the non-exposed. Moreover, using a 450 K epigenome-wide scan and after adjusting by cell type composition and multiple comparisons, 31 CpG sites with significant differences in methylation between the exposed and non-exposed groups were obtained. It was found for the first time, that the striking and significant locus that is most differentially methylated is designated to the *ADM* gene, and is hypermethylated.

The present study has characteristics that need to be considered. At term and appropriate for gestational age newborns were included, avoiding the effect of intrauterine growth restriction. Cotinine, the most sensitive biomarker to evaluate tobacco exposure [19], was measured in maternal plasma samples and umbilical cord blood in newborns. However, due to its short half-life in blood during pregnancy, it reflects only recent maternal smoking. Therefore questionnaires were employed to confirm that the smoking habit was maintained prior to and throughout the pregnancy.

The epigenetic-wide DNA profile of whole blood from umbilical cord was assessed by using a 450 K Illumina platform with the innovative Houseman methodology to exclude potential confounders [13]. A previous study, which used the Illumina 450 K platform [20] measured DNA methylation in unfractionated PBLs with respect to cell variability in whole blood samples representing the methylation of a complex cellular mixture. The method used in the present study normalizes the DNA methylation data with respect to cell variability in whole blood samples [13]. This approach has already successfully examined the association between inferred cell mixture in umbilical cord blood and in *utero* arsenic exposure [8]. Even though previous studies have analysed DNA methylation abnormalities in a larger population of smoker mothers, they did not apply the Houseman correction, as used in the present study.

DNA methylation patterns are essential for the growth and maintenance of tissue-specific expression profiles in different cell types during development, and these patterns become set during in utero development [21,22]. This is true both in the fetus itself and in the placenta, where changes to the appropriate methylation patterning have been linked to adverse placental morphology and birth outcomes [23,24]. A lower degree of placental global DNA methylation in association with exposure to particulate air pollution during early pregnancy has been previously assessed [25], demonstrating how environmental conditions affect gene specific DNA methylation and gene expression patterns during the fetal period. Few human studies have examined epigenetic alterations in relation to maternal smoking during pregnancy [9-11]. These studies have used a [3H]-methyl acceptance assay of global methylation [9], long interspersed nuclear element-1 (LINE-1) and short interspersed element (AluYb8) [10], or differential methylation at cytosine–guanine dinucleotide (CpG) sites [11]. Maternal tobacco use during pregnancy has also been associated with global (LINE-1 and AluYb8) and CpG-site specific methylation in human placenta [11] and buccal cells from children [12]. Genes with a differing methylation pattern, reduced in the aryl hydrocarbon receptor repressor gene (*AHRR*) and increased in the cytochrome P450, family 1, subfamily A, polypeptide 1 *(CYP1A1)*, have been described in adult smokers [26] and in newborns exposed to inutero tobacco [20]. DNA methylation is generally associated with gene transcription repression, and so by consequence, a low level of methylation, usually in the promoter region of the genes, allows for a higher expression of the encoded protein. On the other hand, high methylation implies a lower expression. In the present study, no different methylation pattern in these two genes has been detected, possibly due to the sample size.

The results of the present study link the *ADM* hypermethylation to maternal smoking. This gene was identified in 1993 as a peptide with vasodilatation function, but since then has been linked to a wide range of biological actions including cell growth, regulation of hormone secretion and natriuresis [27]. Moreover, the *ADM* has been found to be related to chronic diseases such as obesity and comorbidities such as diabetes, atherosclerosis and coronary heart disease [28,29], all of which have been associated with in utero tobacco exposure [3]. This observation increases the importance of the *AHRR/ADM* pathway as a biomarker of early tobacco effects. Even though *ADM* was not previously associated with maternal smoking and DNA methylation, a recent study has demonstrated that *ADM* significantly contributes to the carcinogenic effect of *AHRR* and tobacco [30]. The study found that *ADM* and *AHRR* were co-upregulated in lung tumour patients and the *ADM* expression is dependent on tobacco exposure. Aryl hydrocarbon receptor repressor gene directly regulates *ADM* expression under normal physiologic conditions but may also play a role during the early stages of tumour genesis in the lung. Moreover, *ADM* is also a key peptide in cardiovascular regulation [27-29]. Even though the long-term significance of the *ADM* alteration observed in the present study is unknown, the relationship between tobacco exposure and elevated blood pressure, type 2 diabetes, and obesity has been previously demonstrated [3].

Other loci were identified with higher or lower methylation when compared between the exposed and non-exposed groups. These can mediate their effects by: a) regulation of gene transcription directly or indirectly (*FOXP4, ZNF827, AIRE, NAV2, POU2F, ZBTB45, PPP1R15, ANKIRIN1, PLD3, RSU1 PDE4D*); and b) protein synthesis and processing (*RPS2, RPL27, SPSB4, CLEF6, RPS25 and RPL27*). The potential impact of changes found in DNA methylation can modify gene expression in a small proportion, but these changes along life can result in important effects. Prospective studies of children exposed to tobacco in which the DNA methylation pattern has been assessed can help to clarify the impact of these alterations on the long life effect.

## Conclusions

In utero tobacco exposure, even in the absence of fetal growth restriction, may alter the epigenome, with global DNA hypomethylation. Specific genes targeted by tobacco exposure can be identified. The genes found indicate that the potential cellular mechanisms modified by tobacco exposure are numerous and involve different overall regulation mechanisms. The DNA status at birth can therefore be used as a biomarker of prenatal insults. While studies of the cord blood give an indication of epigenetic changes experienced in utero, the question remains if this in utero exposure persists into childhood and adulthood. Considering the possibility to reverse epigenetic modifications, a window of opportunity exists to modify the programmed chronic disease. Prospective studies are necessary to assess the impact of epigenomic changes at birth, on health and disease.
